# Progression to dementia in memory clinic patients with mild cognitive impairment and normal β-amyloid

**DOI:** 10.1186/s13195-019-0557-1

**Published:** 2019-12-05

**Authors:** Anna Rosenberg, Alina Solomon, Vesna Jelic, Göran Hagman, Nenad Bogdanovic, Miia Kivipelto

**Affiliations:** 1 Department of Neurology, Institute of Clinical Medicine, University of Eastern Finland, Kuopio, Finland; 2 Division of Clinical Geriatrics, Centre for Alzheimer Research, Department of Neurobiology, Care Sciences and Society, Karolinska Institutet, Stockholm, Sweden; 3 Clinic for Cognitive Disorders, Theme Aging, Karolinska University Hospital-Huddinge, Stockholm, Sweden; 4 Institute of Public Health and Clinical Nutrition, University of Eastern Finland, Kuopio, Finland; 5 The Ageing Epidemiology Research Unit, School of Public Health, Imperial College London, London, UK

**Keywords:** Mild cognitive impairment, Alzheimer’s disease, Dementia, Disease progression, Prognosis, Biomarkers, Cerebrospinal fluid

## Abstract

**Background:**

Determination of β-amyloid (Aβ) positivity and likelihood of underlying Alzheimer’s disease (AD) relies on dichotomous biomarker cut-off values. Individuals with mild cognitive impairment (MCI) and Aβ within the normal range may still have a substantial risk of developing dementia, primarily of Alzheimer type. Their prognosis, as well as predictors of clinical progression, are not fully understood. The aim of this study was to explore the associations of cerebrospinal fluid (CSF) biomarkers (Aβ42, total tau, phosphorylated tau) and other characteristics, including modifiable vascular factors, with the risk of progression to dementia among patients with MCI and normal CSF Aβ42.

**Methods:**

Three hundred eighteen memory clinic patients with CSF and clinical data, and at least 1-year follow-up, were included. Patients had normal CSF Aβ42 levels based on clinical cut-offs. Cox proportional hazard models with age as time scale and adjusted for sex, education, and cognition (Mini-Mental State Examination) were used to investigate predictors of progression to dementia and Alzheimer-type dementia. Potential predictors included CSF biomarkers, cognitive performance (verbal learning and memory), apolipoprotein E (*APOE*) ε4 genotype, medial temporal lobe atrophy, family history of dementia, depressive symptoms, and vascular factors, including the Cardiovascular Risk Factors, Aging and Dementia (CAIDE) risk score. Predictive performance of patient characteristics was further explored with Harrell C statistic.

**Results:**

Lower normal Aβ42 and higher total tau and phosphorylated tau were associated with higher dementia risk, and the association was not driven by Aβ42 values close to cut-off. Additional predictors included poorer cognition, *APOE* ε4 genotype, higher systolic blood pressure, and lower body mass index, but not the CAIDE dementia risk score. Aβ42 individually and in combination with other CSF biomarkers improved the risk prediction compared to age and cognition alone. Medial temporal lobe atrophy or vascular factors did not increase the predictive performance.

**Conclusions:**

Possibility of underlying AD pathology and increased dementia risk should not be ruled out among MCI patients with CSF Aβ42 within the normal range. While cut-offs may be useful in clinical practice to identify high-risk individuals, personalized risk prediction tools incorporating continuous biomarkers may be preferable among individuals with intermediate risk. The role of modifiable vascular factors could be explored in this context.

## Background

Mild cognitive impairment (MCI) is a heterogeneous condition characterized by subjective cognitive complaints and objectively measured mild impairment in at least one cognitive domain [1]. The cumulative risk of progression to dementia in MCI has been estimated to range from approximately 22% (community-based studies) to 39% (memory clinics), with the majority of individuals developing Alzheimer-type dementia [2]. To accurately identify individuals with underlying Alzheimer’s disease (AD) early, at the MCI or even asymptomatic stage, several sets of diagnostic research criteria were proposed [3–9]. All criteria build upon biomarkers of AD neuropathology, combining them in different ways to classify individuals based on the probability of AD [3–9]. β-amyloid 1–42 (Aβ42) and its deposition in insoluble plaques in the brain is considered a pathological hallmark of AD [10]. Thus, diagnostic research criteria for AD underline the importance of biomarkers reflecting the accumulation of Aβ42, namely decreased levels of Aβ42 in the cerebrospinal fluid (CSF) and increased uptake of Aβ42-specific tracers in positron emission tomography (PET) [3–9]. The most recent criteria, the National Institute on Aging–Alzheimer’s Association (NIA-AA) Research Framework, propose that amyloid positivity, regardless of cognitive performance or other biomarker evidence, could be sufficient for classifying a person as being “in the Alzheimer’s continuum” [9].

Determination of amyloid positivity currently relies on cut-off values. CSF cut-offs used in clinical practice vary between laboratories/clinics, and they are generally based on comparisons between healthy individuals and those with AD dementia diagnosis. Other cut-offs have been proposed by, e.g., examining the concordance of CSF Aβ42 with amyloid PET [11] or utilizing data-driven modeling [12]. Nevertheless, the amyloid positive versus negative dichotomy may not capture the full continuum of AD and dementia risk. In fact, 10–40% of MCI individuals with normal CSF Aβ42 may ultimately develop AD dementia [13–17], and CSF Aβ42, as well as the other core AD biomarkers total tau (t-tau) and phosphorylated tau (p-tau), have been associated with an increased risk of progression to AD dementia among memory clinic patients with normal levels of CSF Aβ42 [18].

As studies are scarce, and previous findings may not always be generalizable to other populations or settings, more evidence is needed for the role of CSF Aβ42 and other biomarkers in predicting progression to dementia among individuals with CSF Aβ42 within the normal range. Moreover, it is not fully clear if other characteristics, such as modifiable vascular and lifestyle-related factors, are associated with the risk of progression in MCI patients with normal Aβ42 levels. Such information might help identify a window of opportunity for secondary prevention in this patient population and inform lifestyle-based and vascular prevention trials—which may not select subjects based on their amyloid status—of potential new targets.

The aim of this study was to investigate whether (1) levels of CSF Aβ42, t-tau, and p-tau and (2) other characteristics, including modifiable vascular factors, were associated with the risk of progression to dementia/AD dementia among memory clinic patients with MCI and normal CSF Aβ42 levels.

## Methods

### Study population

The study included 318 patients diagnosed with MCI at the Karolinska University Hospital memory clinic in Huddinge, Sweden, during 2007–2014, who consented to have their data included in the clinic’s research database. Criteria used to identify individuals in the database for the present study were as follows: at least 1 year of follow-up, availability of baseline CSF and other data relevant for the study, and normal CSF Aβ42 levels based on the cut-offs employed at the clinic. The study was approved by the Regional Ethical Review Board in Stockholm, and written informed consent was obtained from all patients.

Routine assessments at the memory clinic consisted of a physical and neurological examination, thorough review of medical history, Mini-Mental State Examination (MMSE) [19] and comprehensive neuropsychological testing, routine blood tests, CSF sampling to measure AD biomarkers (Aβ42, t-tau, p-tau), brain imaging (structural magnetic resonance imaging, MRI or computed tomography, CT), and other assessments, depending on the patient’s clinical presentation [20]. Diagnoses were made on average within 2 months from the beginning of the assessment period by consensus in multidisciplinary meetings of the clinic staff using all available clinical data, including CSF biomarker data, in an unblinded manner. MCI was diagnosed using the consensus criteria for MCI which require the presence of both subjective and objective cognitive impairment involving one or more cognitive domains, but no impairment of activities of daily living and no dementia [21]. Objective cognitive impairment was defined as a test performance of 1.5 SD below what is expected based on age and education. Dementia diagnoses were made according to the Diagnostic and Statistical Manual of Mental Disorders, 4^th^ edition (DSM-IV) [22] criteria, and etiology was diagnosed using the National Institute of Neurological and Communicative Disorders and Stroke–Alzheimer’s Disease and Related Disorders Association (NINCDS-ADRDA) criteria by McKhann et al. [23] for AD, National Institute of Neurological Disorders and Stroke–Association Internationale pour la Recherche et l’Enseignement en Neurosciences (NINDS-AIREN) [24] criteria for vascular dementia, criteria by Neary et al. [25] for frontotemporal dementia, and the Movement Disorder Society Task Force [26] criteria for Parkinson’s disease dementia, as previously reported [27, 28]. The necessity and frequency of follow-up visits were based on the clinician’s judgment as per local routine clinical practice. Follow-up data in the present study were collected until April 2018. Main outcome in this study was progression to any dementia, and progression to AD dementia was also investigated.

### CSF biomarkers

CSF samples were collected by standard lumbar puncture done between the L3/L4 or L4/L5 intervertebral space with a 25-gauge needle. Samples were collected in polypropylene tubes and centrifuged within 2 h. CSF Aβ42, t-tau, and p-tau concentrations were measured with commercially available sandwich enzyme-linked immunosorbent assays (Innogenetics, Ghent, Belgium) [29]. CSF Aβ42 positivity was determined based on the reference values provided by the laboratories conducting the analyses, and employed in clinical practice at the memory clinic, as previously reported [27, 30–32] (cut-off < 450 pg/ml defined by the Karolinska University Hospital in Stockholm, Sweden, until 2011; cut-off < 550 pg/ml defined by the Clinical Neurochemistry Laboratory at Sahlgrenska University Hospital in Gothenburg, Sweden, from 2012 onwards). Patients with Aβ42 levels ≥ 450 pg/ml (first visit during 2007–2011) or ≥ 550 pg/ml (first visit in 2012 or later) were considered to have normal CSF Aβ42 levels. Cut-off values for abnormal t-tau and p-tau were ≥ 400 pg/ml and ≥ 80 pg/ml, respectively [30].

As there is increasing evidence suggesting that the currently used clinical cut-offs for CSF Aβ42 positivity may be too conservative due to, e.g., upward drift in Aβ42 values over time in certain assays [33, 34], and more lenient cut-offs may predict underlying amyloid pathology more accurately [11, 12], we performed sensitivity analyses in a subsample of patients with CSF Aβ42 levels > 696 pg/ml (*N* = 195). This cut-off for MCI patients was proposed by Bertens et al. [12] in a Dutch memory clinic population using a data-driven approach. The Dutch clinical cut-off, 550 pg/ml, was comparable to the one used at the Karolinska University Hospital memory clinic.

### MRI and CT assessment

Brain MRI or CT scans were performed in connection to the first visit at the memory clinic or as part of the general practitioner’s initial assessment prior to the referral. MRI visual assessments were performed based on T1-weighted images, and medial temporal lobe atrophy (MTA) was rated using the Scheltens scale [35] ranging from 0 (no atrophy) to 4 (severe atrophy). Where applicable, MTA rating was performed also on the CT scans (54 of the 237 patients with available brain imaging data). MTA was visually assessed for right and left hemisphere separately, and the mean score was used in the present study.

### *APOE* genotyping

DNA was extracted from blood leucocytes using standard methods polymerase chain reaction and HhaI digestion, and apolipoprotein E (*APOE*) genotype was determined by a microsequencing method on microtiter plates (AffiGene ApoE, Sangtec Medical, Bromma, Sweden), as previously described [29, 30].

### Other clinical characteristics

Patients’ demographic and clinical characteristics, including age, sex, years of formal education, cognitive test scores (MMSE, Rey Auditory Verbal Learning Test (RAVLT) immediate and delayed recall [36]), and information about medications and medical history (hypertension, hyperlipidemia, diabetes) were obtained from medical records from the baseline visit at the memory clinic. Additional data collected from the medical records included the presence of depressive symptoms (measured on Cornell Scale for Depression in Dementia [37]), systolic and diastolic blood pressure, smoking habits, and height and weight. Body mass index (BMI) was calculated by dividing the weight in kilograms by the squared height in meters. Self-reported family history of any type of dementia was considered positive when there was at least one affected first-degree relative.

The Cardiovascular Risk Factors, Aging and Dementia (CAIDE) risk score [38] was calculated using demographic and clinical data collected from the medical records (Additional file 1: Table S1). As data about lifestyle, including physical activity, were not routinely collected at the memory clinic, the physical activity component was excluded from the CAIDE risk score [39]. For elevated total cholesterol, two points were given if the patient had hyperlipidemia (diagnosis and treatment with any lipid-lowering drug). For elevated systolic blood pressure (SBP), two points were given if the patient either had hypertension (diagnosis and treatment with any antihypertensive drug), or SBP measured at the first visit was elevated (> 140 mmHg). The CAIDE risk score version used in the present study ranged from 0 to 14 (version with *APOE* 0–17), with higher values indicating higher risk of dementia.

### Statistical analysis

Baseline demographic and clinical characteristics were compared between patients who progressed to dementia and those who did not with *t* tests and chi-square tests, as appropriate. Associations between individual baseline characteristics and risk of progression to dementia/AD dementia were analyzed with Cox proportional hazard models with age as time scale. Models included additionally sex, education, and baseline MMSE as covariates. Results are reported as hazard ratios (HR) and 95% confidence intervals (CI). Zero-skewness log-transformation followed by z-transformation was applied to CSF biomarkers to obtain hazard ratios per SD and to compare the associations with dementia/AD dementia between different biomarkers.

We further estimated the predictive performance (Harrell C statistic) of all individual baseline characteristics showing significant associations with the risk of progression to any dementia in the univariate analysis (*p* < 0.10). Harrell C statistic of 0.5 indicates no predictive value, whereas 1.0 indicates complete prediction, on a scale from 0 to 1. We compared the Harrell C statistic of a basic model including only age and cognitive performance (RAVLT delayed recall score) with models expanded with additional predictors. All analyses were conducted with Stata software version 14, and level of statistical significance was set to *p* < 0.05.

## Results

Baseline characteristics of the study population are shown in Table 1. Patients’ mean age was 64.8 years, and 174 (54.7%) were women. Patients had on average 11.7 years of education, and their mean MMSE score was 27.1 points. In total, 50.3% (86 out of 171) of the patients with available *APOE* genotype data were ε4 carriers, and 38.9% (119 out of 306) had a family history of dementia. CSF t-tau and p-tau levels were considered abnormal in 92 (29.0%) and 60 (18.9%) patients, respectively. Of the 236 patients for whom all biomarkers were available, 135 (57.2%) could be classified as having evidence for neurodegeneration in the absence of definitely abnormal Aβ, i.e., at least one abnormal marker of neurodegeneration (CSF t-tau, p-tau, MTA).

**Table 1:** Baseline characteristics of the study population, by outcome (progression to any dementia)

Characteristics	Data available	All (*N* = 318)	Progression to dementia (*N* = 121)	No progression to dementia (*N* = 197)	*P* value
**Demographics**					
Age, years	318	64.8 (9.1)	67.9 (8.3)	63.0 (9.1)	*< 0.001*
Female	318	174 (54.7%)	64 (52.9%)	110 (55.8%)	0.61
Education, years	293	11.7 (3.7)	11.7 (3.5)	11.7 (3.9)	0.97
Follow-up time, years	318	2.8 (1.9)	2.9 (1.9)	2.8 (1.9)	0.83
**Cognition**					
MMSE score	315	27.1 (2.5)	27.0 (2.3)	27.1 (2.7)	0.72
RAVLT, immediate recall score	215	34.6 (9.8)	30.6 (8.4)	37.5 (9.7)	*< 0.001*
RAVLT, delayed recall score	235	5.6 (3.3)	4.2 (2.9)	6.5 (3.3)	*< 0.001*
**Biomarkers**					
CSF Aβ42, pg/ml	318	849.1 (293.6)	703.7 (222.5)	938.5 (296.7)	*< 0.001*
CSF t-tau, pg/ml	317	335.5 (184.9)	418.0 (193.5)	284.5 (159.7)	*< 0.001*
CSF p-tau, pg/ml	317	58.0 (25.1)	65.7 (22.8)	53.3 (25.4)	*< 0.001*
Abnormal t-tau (≥ 400 pg/ml)	317	92 (29.0%)	55 (45.5%)	37 (18.9%)	*< 0.001*
Abnormal p-tau (≥ 80 pg/ml)	317	60 (18.9%)	31 (25.6%)	29 (14.8%)	*0.02*
CSF Aβ42/t-tau ratio	317	3.4 (2.0)	2.2 (1.4)	4.1 (2.0)	*< 0.001*
CSF Aβ42/p-tau ratio	317	18.0 (10.4)	12.5 (6.9)	21.4 (10.7)	*< 0.001*
MTA score	237	1.1 (0.8)	1.3 (0.7)	1.0 (0.8)	*0.001*
MTA score > 1	237	84 (35.4%)	45 (46.9%)	39 (27.7%)	*0.002*
**Vascular factors**					
Systolic BP, mmHg	245	143.5 (19.2)	148.4 (21.7)	140.6 (17.1)	*0.002*
Diastolic BP, mmHg	245	83.4 (10.5)	84.7 (10.5)	82.6 (10.5)	0.13
BMI, kg/m^2^	205	26.0 (4.0)	25.5 (4.0)	26.3 (4.0)	0.23
Current smoking	287	41 (14.3%)	15 (13.5%)	26 (14.8%)	0.77
Hypertension	318	130 (40.9%)	54 (44.6%)	76 (38.6%)	0.29
Hyperlipidemia	318	93 (29.3%)	40 (33.1%)	53 (26.9%)	0.24
Diabetes	318	53 (16.7%)	20 (16.5%)	33 (16.8%)	0.96
CAIDE risk score	174	7.5 (2.5)	7.9 (2.1)	7.4 (2.6)	0.18
CAIDE risk score with *APOE*	91	9.4 (2.4)	9.8 (2.4)	9.2 (2.4)	0.22
**Other factors**					
*APOE* ε4 carrier	171	86 (50.3%)	44 (65.7%)	42 (40.4%)	*0.001*
Family history of dementia	306	119 (38.9%)	47 (39.8%)	72 (38.3%)	0.79
Cornell score	248	6.1 (4.7)	5.2 (4.2)	6.6 (4.9)	*0.02*

Data are mean (SD) or *N* (%). *P* values are shown for comparisons between patients who developed/did not develop dementia. *P* values < 0.05 are italicized. Hypertension and hyperlipidemia were defined as diagnosis of hypertension/hyperlipidemia and treatment with any antihypertensive/lipid-lowering drug. Family history of dementia included at least one affected first-degree relative. *APOE* apolipoprotein E, *Aβ42* β-amyloid 1–42, *BMI* body mass index, *BP* blood pressure, *CAIDE* Cardiovascular Risk Factors, Aging and Dementia Study, *CSF* Cerebrospinal fluid, *MMSE* Mini-Mental State Examination, *MTA* medial temporal lobe atrophy, visual rating, *RAVLT* Rey Auditory Verbal Learning Test, *p-tau* tau phosphorylated at threonine 181, *t-tau* total tau

During a mean follow-up period of 2.8 years (SD 1.9, range 1–10 years), 121 patients (38.1%) progressed to any dementia, with most cases being AD dementia (*N* = 91). Other dementias included unspecified dementia (*N* = 11), vascular dementia (*N* = 10), frontotemporal dementia (*N* = 6), and Parkinson’s disease dementia (*N* = 3). Patients with evidence for neurodegeneration progressed to dementia more often than those with normal biomarkers of neurodegeneration (70 out of 135; 51.9% vs. 26 out of 101; 25.7%, *p* < 0.001). Differences in baseline characteristics between patients who progressed to dementia and patients who remained stable or reverted to normal are presented in (Table 1). Patients progressing to dementia were significantly older and performed worse in verbal learning and memory test (RAVLT) than patients who did not show clinical progression. Furthermore, they had significantly lower, but still normal, CSF Aβ42 levels, higher t-tau and p-tau levels, higher MTA score, and lower Cornell score, indicating fewer depressive symptoms. Patients who progressed to dementia were also more often *APOE* ε4 carriers. Vascular risk profile of converters and non-converters was similar, except for SBP which was higher among patients who developed dementia. Similar baseline differences were observed between patients who progressed to AD dementia and those who did not progress to any dementia (Additional file 1: Table S2).

### CSF biomarkers and risk of progression to dementia/AD dementia

Associations of CSF biomarkers with the risk of progression to dementia are presented in Table 2. Among patients with normal CSF Aβ42 levels, higher Aβ42 (HR 0.65, 95% CI 0.52–0.81) was related to a lower risk of dementia, while higher t-tau (HR 2.16, 95% CI 1.70–2.74) and p-tau (HR 1.53, 95% CI 1.25–1.89) were associated with a higher risk of progression to any dementia. Higher Aβ42/t-tau and Aβ42/p-tau ratios were associated with a lower risk of dementia. Similar significant associations were observed in analyses with AD dementia as outcome (Table 2), and in sensitivity analyses including patients classified as having normal CSF Aβ42 levels based on a higher cut-off for amyloid positivity (> 696 pg/ml) (Table 3). Of the three CSF biomarkers, t-tau levels showed the highest HRs for progression to any dementia and AD dementia.

**Table 2:** Association of CSF biomarkers with the risk of progression to any dementia/AD dementia

CSF biomarker	Progression to any dementia, HR (95% CI)	Progression to AD dementia, HR (95% CI)
Aβ42	0.65 (0.52–0.81)	0.55 (0.42–0.72)
t-tau	2.16 (1.70–2.74)	3.28 (2.42–4.46)
p-tau	1.53 (1.25–1.89)	1.99 (1.55–2.56)
Aβ42/t-tau	0.45 (0.35–0.57)	0.28 (0.20–0.40)
Aβ42/p-tau	0.56 (0.45–0.70)	0.41 (0.31–0.54)

HR (hazard ratios) (95% CI) per 1 SD increase in CSF biomarkers are shown from Cox proportional hazard models with age as time scale. Models were adjusted for sex, education, and baseline MMSE score. *AD* Alzheimer’s disease, *Aβ42* β-amyloid 1–42, *CSF* cerebrospinal fluid, *p-tau* tau phosphorylated at threonine 181, *t-tau* total tau

**Table 3:** Association of CSF biomarkers with the risk of progression to any dementia/AD dementia (patients with CSF Aβ42 > 696 pg/ml)

CSF biomarker	Progression to any dementia, HR (95% CI)	Progression to AD dementia, HR (95% CI)
Aβ42	0.58 (0.39–0.87)	0.55 (0.33–0.93)
t-tau	2.36 (1.64–3.38)	4.33 (2.52–7.44)
p-tau	1.43 (1.04–1.96)	1.99 (1.28–3.08)
Aβ42/t-tau	0.38 (0.25–0.56)	0.21 (0.11–0.38)
Aβ42/p-tau	0.55 (0.39–0.79)	0.37 (0.22–0.62)

HR (hazard ratios) (95% CI) per 1 SD increase in CSF biomarkers are shown from Cox proportional hazard models with age as time scale. Models were adjusted for sex, education, and baseline MMSE score. *AD* Alzheimer’s disease, *Aβ42* β-amyloid 1–42, *CSF* cerebrospinal fluid, *p-tau* tau phosphorylated at threonine 181, *t-tau* total tau

### Other characteristics and risk of progression to dementia/AD dementia

Associations of other patient characteristics with the risk of progression to dementia are presented in Table 4. Having lower RAVLT immediate and delayed recall test scores (HR 0.94, 95% CI 0.91–0.96 and HR 0.81, 95% CI 0.75–0.88, respectively) and being *APOE* ε4 carrier (HR 2.03, 95% CI 1.18–3.46) were associated with a higher risk of dementia. Depressive symptoms were associated with a lower risk of progression (HR 0.94, 95% CI 0.89–0.99). Having an MTA score > 1 showed a borderline significant association with dementia risk (HR 1.53, 95% CI 0.99–2.36). Among vascular factors, higher SBP (HR 1.02, 95% CI 1.01–1.03) and lower BMI (HR 0.93, 95% CI 0.86–0.99) were significantly associated with a higher dementia risk. CAIDE dementia risk score, with or without *APOE*, was not associated with an increased dementia risk in this patient population. Similar results were obtained from analyses with AD dementia as outcome (Table 4). However, more pronounced MTA was not significantly associated with risk of progression to AD dementia (HR 1.45, 95% CI 0.88–2.39), and lower BMI showed only a borderline significant association (HR 0.93, 95% CI 0.85–1.00). Associations of patient characteristics with the risk of progression to dementia among patients with CSF Aβ42 > 696 pg/ml are presented in Table 5.

**Table 4:** Association of cognition, vascular factors, and other characteristics with the risk of progression to any dementia/AD dementia

Characteristics	HR (95% CI)	
	Any dementia	AD dementia
**Cognition**		
RAVLT, immediate recall score	0.94 (0.91–0.96)	0.94 (0.91–0.97)
RAVLT, delayed recall score	0.81 (0.75–0.88)	0.82 (0.74–0.90)
**Vascular factors**		
Systolic BP, mmHg	1.02 (1.01–1.03)	1.01 (1.00–1.02)
Diastolic BP, mmHg	1.01 (0.99–1.03)	1.00 (0.97–1.02)
BMI, kg/m^2^	0.93 (0.86–0.99)	0.93 (0.85–1.00)
Current smoking	0.96 (0.54–1.69)	1.05 (0.57–1.95)
Hypertension	1.19 (0.81–1.75)	1.01 (0.64–1.59)
Hyperlipidemia	0.98 (0.65–1.47)	0.90 (0.56–1.45)
Diabetes	0.74 (0.44–1.25)	0.70 (0.38–1.31)
CAIDE risk score	1.00 (0.88–1.14)	0.97 (0.83–1.13)
CAIDE risk score with *APOE*	1.11 (0.91–1.35)	1.07 (0.87–1.33)
**Other factors**		
MTA score > 1	1.53 (0.99–2.36)	1.45 (0.88–2.39)
*APOE* ε4 genotype	2.03 (1.18–3.46)	2.50 (1.36–4.61)
Family history of dementia	1.00 (0.67–1.48)	1.04 (0.66–1.62)
Cornell score	0.94 (0.89–0.99)	0.90 (0.85–0.96)

HR (hazard ratios) (95% CI) are shown from Cox proportional hazard models with age as time scale. Models were adjusted for sex, education, and baseline MMSE score. Hypertension and hyperlipidemia were defined as diagnosis of hypertension/hyperlipidemia and treatment with any antihypertensive/lipid-lowering drug. Family history of dementia included at least one affected first-degree relative. *AD* Alzheimer’s disease, *APOE* apolipoprotein E, *BMI* body mass index, *BP* blood pressure, *CAIDE* Cardiovascular Risk Factors, Aging and Dementia Study, *MTA* medial temporal lobe atrophy, visual rating, *RAVLT* Rey Auditory Verbal Learning Test

**Table 5:** Association of cognition, vascular factors, and other characteristics with the risk of progression to any dementia/AD dementia (patients with CSF Aβ42 > 696 pg/ml)

Characteristics	HR (95% CI)	
	Any dementia	AD dementia
**Cognition**		
RAVLT, immediate recall score	0.92 (0.88–0.97)	0.93 (0.88–0.99)
RAVLT, delayed recall score	0.79 (0.69–0.91)	0.84 (0.72–0.99)
**Vascular factors**		
Systolic BP, mmHg	1.03 (1.01–1.05)	1.02 (0.99–1.04)
Diastolic BP, mmHg	1.01 (0.98–1.05)	0.98 (0.94–1.03)
BMI, kg/m^2^	0.89 (0.80–0.99)	0.95 (0.82–1.11)
Current smoking	1.21 (0.46–3.18)	1.29 (0.37–4.52)
Hypertension	1.28 (0.69–2.39)	1.34 (0.60–2.97)
Hyperlipidemia	0.87 (0.45–1.68)	0.51 (0.21–1.26)
Diabetes	0.68 (0.31–1.46)	0.89 (0.36–2.20)
CAIDE risk score	0.99 (0.82–1.19)	1.03 (0.79–1.34)
CAIDE risk score with *APOE*	1.29 (0.86–1.93)	1.49 (0.81–2.75)
**Other factors**		
MTA score > 1	2.21 (1.09–4.45)	2.60 (1.08–6.29)
*APOE* ε4 genotype	2.07 (0.83–5.17)	3.28 (1.11–9.71)
Family history of dementia	0.62 (0.30–1.29)	0.57 (0.22–1.47)
Cornell score	0.94 (0.87–1.02)	0.85 (0.75–0.97)

HR (hazard ratios) (95% CI) are shown from Cox proportional hazard models with age as time scale. Models were adjusted for sex, education, and baseline MMSE score. Hypertension and hyperlipidemia were defined as diagnosis of hypertension/hyperlipidemia and treatment with any antihypertensive/lipid-lowering drug. Family history of dementia included at least one affected first-degree relative. *AD* Alzheimer’s disease, *APOE* apolipoprotein E, *Aβ42* β-amyloid 1–42, *BMI* body mass index, *BP* blood pressure, *CAIDE* Cardiovascular Risk Factors, Aging and Dementia Study, *CSF* cerebrospinal fluid, *MTA* medial temporal lobe atrophy, visual rating, *RAVLT* Rey Auditory Verbal Learning Test

### Predictive performance of CSF biomarkers and other patient characteristics

Predictive performance of the basic model (age and cognition) and models expanded with additional other predictors (CSF Aβ42 individually and in combination with other CSF biomarkers, MTA, *APOE*, depressive symptoms, SBP, BMI) are shown in Table 6. Harrell C statistic for the basic model ranged from 0.68 to 0.72, depending on the number of patients included in each model (with variations due to missing data). Adding Aβ42 increased Harrell C from 0.69 to 0.73. Together with Aβ42, p-tau and t-tau further improved predictive performance (Harrell C 0.75 for p-tau, 0.74 for Aβ42/p-tau ratio, 0.76 for t-tau and Aβ42/t-tau ratio, and 0.78 for all three CSF biomarkers). Similar results were obtained in sensitivity analyses using a more lenient cut-off for amyloid positivity (Table 7): adding Aβ42 alone to the basic model increased Harrell C from 0.74 to 0.79, and the model including all three CSF biomarkers had the highest predictive performance (0.83).

**Table 6:** Harrell C statistic and model performance in prediction of any dementia/AD dementia

Categories of predictors	*N*	Prediction model	Dementia	AD dementia
			Harrell C	
Basic model vs. CSF biomarkers	234	Age, RAVLT (basic)	0.69	0.69
		Age, RAVLT, Aβ42	0.73	0.75
		Age, RAVLT, Aβ42, p-tau	0.75	0.78
		Age, RAVLT, Aβ42/p-tau	0.74	0.78
		Age, RAVLT, Aβ42, t-tau	0.76	0.81
		Age, RAVLT, Aβ42/t-tau	0.76	0.81
		Age, RAVLT, Aβ42, p-tau, t-tau	0.78	0.83
Basic model vs. MTA	171	Age, RAVLT (basic)	0.71	0.72
		Age, RAVLT, MTA	0.71	0.72
Basic model vs. *APOE* ε4 genotype	133	Age, RAVLT (basic)	0.68	0.68
		Age, RAVLT, *APOE*	0.72	0.72
Basic model vs. SBP	182	Age, RAVLT (basic)	0.69	0.71
		Age, RAVLT, SBP	0.70	0.71
Basic model vs. BMI	141	Age, RAVLT (basic)	0.72	0.71
		Age, RAVLT, BMI	0.74	0.74
Basic model vs. depressive symptoms	183	Age, RAVLT (basic)	0.69	0.72
		Age, RAVLT, Cornell score	0.70	0.74

Predictive performance of the prediction models was explored using the Harrell C statistic (0.5 indicates no predictive value, whereas 1.0 indicates complete prediction, on a scale from 0 to 1). Comparisons of model performance were conducted between models including the same number of patients: basic model (age, RAVLT delayed recall score) and models expanded with additional predictors. *AD* Alzheimer’s disease, *APOE* apolipoprotein E, *Aβ42* β-amyloid 1–42, *BMI* body mass index; *CSF* cerebrospinal fluid, *MTA* medial temporal lobe atrophy, visual rating, *p-tau* tau phosphorylated at threonine 181, *RAVLT* Rey Auditory Verbal Learning Test (delayed recall), *SBP* systolic blood pressure, *t-tau* total tau

**Table 7:** Harrell C statistic and model performance in prediction of any dementia/AD dementia (patients with CSF Aβ42 > 696 pg/ml)

Categories of predictors	*N*	Prediction model	Dementia	AD dementia
			Harrell C	
Basic model vs. CSF biomarkers	140	Age, RAVLT (basic)	0.74	0.74
		Age, RAVLT, Aβ42	0.79	0.79
		Age, RAVLT, Aβ42, p-tau	0.80	0.82
		Age, RAVLT, Aβ42/p-tau	0.78	0.82
		Age, RAVLT, Aβ42, t-tau	0.82	0.87
		Age, RAVLT, Aβ42/t-tau	0.81	0.86
		Age, RAVLT, Aβ42, p-tau, t-tau	0.83	0.88
Basic model vs. MTA	101	Age, RAVLT (basic)	0.74	0.75
		Age, RAVLT, MTA	0.77	0.78
Basic model vs. *APOE* ε4 genotype	72	Age, RAVLT (basic)	0.73	0.71
		Age, RAVLT, *APOE*	0.77	0.78
Basic model vs. SBP	109	Age, RAVLT (basic)	0.73	0.75
		Age, RAVLT, SBP	0.77	0.78
Basic model vs. BMI	82	Age, RAVLT (basic)	0.78	0.82
		Age, RAVLT, BMI	0.80	0.83
Basic model vs. depressive symptoms	105	Age, RAVLT (basic)	0.74	0.78
		Age, RAVLT, Cornell score	0.77	0.81

Predictive performance of the prediction models was explored using the Harrell C statistic (0.5 indicates no predictive value, whereas 1.0 indicates complete prediction, on a scale from 0 to 1). Comparisons of model performance were conducted between models including the same number of patients: basic model (age, RAVLT delayed recall score) and models expanded with additional predictors. *AD* Alzheimer’s disease, *APOE* apolipoprotein E, *Aβ42* β-amyloid 1–42, *BMI* body mass index, *CSF* cerebrospinal fluid, *MTA* medial temporal lobe atrophy, visual rating, *p-tau* tau phosphorylated at threonine 181, *RAVLT* Rey Auditory Verbal Learning Test (delayed recall), *SBP* systolic blood pressure, *t-tau* total tau

*APOE* ε4 genotype increased the predictive performance of the basic model (increase in Harrell C from 0.68 to 0.72), similarly to Aβ42, but depressive symptoms, MTA, or vascular factors did not substantially improve the predictive performance of the basic model (Table 6). Similar results were obtained with AD dementia as outcome.

## Discussion

This study explored the associations of CSF AD biomarkers and other patient characteristics with progression to dementia among memory clinic patients with MCI and normal CSF Aβ42 levels. Lower Aβ42 levels within the normal range, as well as higher t-tau and p-tau levels, were significantly associated with a higher risk of progression to any dementia and AD dementia in this population. Additional predictors were poorer cognitive performance, *APOE* ε4 genotype, higher SBP, and lower BMI. No association was observed between the CAIDE dementia risk score and risk of progression. Aβ42, both alone and in combination with t-tau and p-tau, improved the prediction of progression to dementia compared to age and cognitive performance alone, whereas vascular factors did not substantially increase the predictive performance.

According to the current diagnostic research criteria for AD [5–9], patients with MCI and normal CSF Aβ42 levels would be assumed to have an underlying non-AD pathology. A range of non-AD-related processes, such as primary age-related tauopathy, hippocampal sclerosis, cerebrovascular disease, argyrophilic grain disease, and TDP-43 encephalopathy, could contribute to neurodegeneration in this population [40–42]. While in previous studies the rate of clinical progression was consistently lower among MCI patients with evidence for neurodegeneration in the absence of definitely abnormal amyloid than among those with abnormal markers of both amyloid and neurodegeneration, a significant number of individuals still developed AD dementia during a fairly short follow-up period [13–17]. In line with these findings, we observed that, despite having normal CSF Aβ42 levels, a high proportion of MCI patients progressed to dementia (primarily of Alzheimer type).

Our results indicating that lower Aβ42 levels within the normal range were significantly associated with an increased risk of progression are also supported by previous findings [18]. It has been unclear whether the effect is primarily driven by Aβ42 values just above the cut-off value [13, 18]; however, we observed a similar pattern in a sensitivity analysis with a more lenient cut-off value for amyloid positivity. This suggests that simply increasing cut-off values might not be sufficient to optimally distinguish future converters from individuals who remain stable or develop non-AD dementia. As the clinical impact of amyloid accumulation and threshold for cognitive decline and disease progression may vary among individuals, due to, e.g., cognitive reserve [43] or mixed pathologies (e.g., vascular) [44], treating biomarkers as continuous may be preferred in certain subgroups of MCI patients. A PET study by Farrell et al. [45] showed that continuous measures reflecting amyloid deposition may provide more detailed information about the predicted rate of cognitive decline than the dichotomized amyloid status. The study focused, however, on cognitively healthy individuals and change in cognition, rather than clinical progression. Also, the effect appeared to be limited to amyloid-positive individuals. Among amyloid-negative individuals, preliminary evidence from recent longitudinal PET studies suggests that amyloid accumulation is associated with cognitive decline [46, 47], whereas baseline amyloid burden is not [46]. Again, these studies did not investigate clinical progression as an outcome, but Landau et al. [47] reported the numbers of individuals who converted to amyloid positive and progressed to MCI or AD. Interestingly, very little overlap was observed between these groups. As these studies did not include patients with MCI, the magnitude and impact of change in amyloid levels at the MCI stage remains unclear.

In our MCI population with CSF Aβ42 levels within the normal range, *APOE* ε4 showed similar predictive value to that of Aβ42 for short-term progression to dementia, suggesting that *APOE* genotyping could potentially be incorporated in the risk assessment in this patient population. *APOE* ε4 genotype has previously been linked to an increased risk of conversion from MCI to dementia, but studies have primarily focused on heterogeneous MCI populations, without stratifying by amyloid pathology [48, 49]. CSF t-tau and p-tau were also associated with a higher risk of progression to dementia/AD dementia in our study. Similar findings were previously reported in patients with normal Aβ42 levels and MCI, but not subjective cognitive impairment [18]. As shown before, neuronal injury biomarkers predict disease progression and correlate well with cognitive deterioration among MCI patients [10]. In our study, however, we did not observe a significant association between visually rated MTA and risk of progression to dementia, and MTA did not substantially increase the predictive performance of age and cognition alone. A measure of hippocampal volume, which may have more predictive value [50, 51], was not available for this study.

Among other patient characteristics, depressive symptoms were associated with a lower risk of dementia/AD dementia. Rather than reflecting a protective effect, our results may indicate that depression was a common underlying cause of MCI, as reported before [52]. Vascular factors, such as smoking, hypertension, hyperlipidemia, diabetes, and the CAIDE dementia risk score, were not associated with an increased risk of dementia in this MCI population with normal CSF Aβ42 levels. Lower BMI and higher SBP increased the risk of dementia/AD dementia, but did not markedly improve the predictive performance compared to age and cognition alone. While vascular and lifestyle-related risk factors have been shown to increase the risk of subsequent dementia in midlife [53], their association with cognitive decline and dementia might be less straightforward at older ages and during shorter follow-ups [54, 55]. Factors such as blood pressure, BMI, or cholesterol have been reported to decline after midlife in individuals who develop dementia later on [56–58], complicating their use as potential predictors, especially in individuals with cognitive impairment. Indeed, our earlier study indicated that the predictive performance of the CAIDE dementia risk score, developed for long-term prediction based on a midlife risk profile, was limited among memory clinic patients with cognitive complaints [20]. With regard to individual modifiable risk factors, a meta-analysis investigating predictors of progression from MCI to dementia reported a protective effect for higher BMI, which is consistent with our findings [59]. Diabetes and hypertension were also associated with a higher risk of progression to AD dementia, while smoking and hypercholesterolemia were not [59]. In contrast, in a large multicenter memory clinic study of MCI patients [52], hypertension, hypercholesterolemia, diabetes, obesity, and smoking did not influence the rate of cognitive decline and disease progression. This effect did not vary across the International Working Group-2 (IWG-2) and NIA-AA criteria, i.e., it did not seem to depend on the presence or absence of amyloid pathology.

Nevertheless, given the associations we observed between lower BMI, higher SBP, and increased dementia risk in this study, the potential role of modifiable vascular factors in subgroups of memory clinic patients with MCI needs to be further studied. Modifiable vascular/lifestyle factors could be relevant targets for preventive strategies, especially in memory clinic patients with normal Aβ42 levels who may not be eligible for amyloid-focused pharmacological dementia prevention trials.

### Strengths and limitations

This study included a large sample of well-characterized memory clinic patients with normal CSF Aβ42 levels, which allowed us to investigate a broad range of different biomarkers and clinical characteristics in relation to subsequent dementia development. As data were collected retrospectively from medical records, detailed information on lifestyle factors was not available. Some variables investigated in this study were only available for a subset of patients, potentially limiting the statistical power of the analyses. Due to missing data, some variables were also excluded from the analyses (e.g., cognitive tests for non-memory domains, Fazekas score for white matter lesions).

As in most clinic-based studies, there was considerable variation in follow-up time and number of visits, and due to the fairly short mean follow-up time of approximately 3 years, the possibility that some individuals are slow progressors and develop dementia at a later stage cannot be excluded. Also, potential circularity cannot be fully ruled out, as clinicians were not blinded to CSF biomarkers. However, CSF was routinely collected at the clinic from all referred patients without contraindications for lumbar puncture, not just those with suspected AD pathology. Also, the study population included all patients with a clinical diagnosis of MCI and normal CSF Aβ42 as per local cut-offs, regardless of clinicians’ notes on MCI subtype or potential underlying causes. Another potential limitation is the upward drift over time in CSF Aβ42 concentrations and cutpoints for normality/abnormality in broadly used assays [33, 34]. To address this issue, we performed sensitivity analyses with a higher cut-off for amyloid positivity and obtained largely comparable results.

Finally, due to differences in patient populations and clinic procedures, our results may not be generalizable to other memory clinics, settings, or populations. As the predictive performance of biomarkers may be influenced by patient age [12, 50, 60], it is noteworthy that the patient population at the Karolinska University Hospital memory clinic is fairly young (mean age of all examined patients 63 years; in this study 65 years) [61]. The patient population in this study was also highly educated and ethnically relatively homogenous, indicating a need for studies in diverse populations and other geographical settings.

## Conclusions

Among memory clinic patients with MCI and normal CSF Aβ42 levels, all three CSF biomarkers Aβ42, t-tau, and p-tau were key predictors of progression to dementia/AD dementia. The association between Aβ42 and clinical progression did not seem to be driven by Aβ42 levels close to the cut-off values. The possibility of underlying AD pathology and risk of progression to dementia should thus not be completely ruled out among MCI patients when Aβ42 levels are within the normal range. While cut-offs may be useful in clinical practice to identify high-risk individuals, establishing an accurate prognosis for individuals with an intermediate risk of progression requires alternative approaches. Simply adjusting the current cut-offs for “normal Aβ42” may not be the optimal solution, but developing more complex personalized risk prediction tools including continuous CSF biomarkers may be preferable [50, 51]. In this context, the role of modifiable vascular/lifestyle factors in different subgroups of MCI patients should be further explored.

## Supplementary information

**Additional file 1: Table S1.** CAIDE risk scores calculated in the present study. **Table S2.** Baseline characteristics of the study population, by outcome (progression to AD dementia).

## Data Availability

The datasets generated and/or analyzed during the current study are not publicly available due to ethics legislation in Sweden. For more information, please contact Miia Kivipelto, miia.kivipelto@ki.se.

## Abbreviations

AD: Alzheimer’s disease
APOE: Apolipoprotein E
Aβ: β-amyloid
Aβ42: β-amyloid 1–42
BMI: Body mass index
BP: Blood pressure
CAIDE: Cardiovascular Risk Factors, Aging and Dementia Study
CI: Confidence interval
CSF: Cerebrospinal fluid
CT: Computed tomography
DSM-IV: Diagnostic and Statistical Manual of Mental Disorders, 4^th^ edition
HR: Hazard ratio
IWG-2: International Working Group-2
MCI: Mild cognitive impairment
MMSE: Mini-Mental State Examination
MRI: Magnetic resonance imaging
MTA: Medial temporal lobe atrophy
NIA-AA: National Institute on Aging–Alzheimer’s Association
NINCDS-ADRDA: National Institute of Neurological and Communicative Disorders and Stroke-Alzheimer’s Disease and Related Disorders Association
NINDS-AIREN: National Institute of Neurological Disorders and Stroke–Association Internationale pour la Recherche et l’Enseignement en Neurosciences
PET: Positron emission tomography
p-tau: Tau phosphorylated at threonine 181
RAVLT: Rey Auditory Verbal Learning Test
SBP: Systolic blood pressure
t-tau: Total tau
