# Supplementary material for: Progression to dementia in memory clinic patients with mild cognitive impairment and normal β-amyloid
Source: Alzheimers Res Ther. 2019 Dec 5;11:99. doi: 10.1186/s13195-019-0557-1 (PMC6896336; doi:10.1186/s13195-019-0557-1)
Supplement: Supplementary file 1 — Additional file 1: Table S1. CAIDE risk scores calculated in the present study. Table S2. Baseline characteristics of the study population, by outcome (progression to AD dementia). [file 13195_2019_557_MOESM1_ESM.docx]

**Supplementary Table 1.** CAIDE risk scores calculated in the present study

| **Risk factor** | **CAIDE risk score** | |
| --- | --- | --- |
|  | **Without APOE** | **With APOE** |
| **Age in years**  <47  47–53  >53 | 0  3  4 | 0  3  5 |
| **Years of formal education**  ≥10  7–9  <7 | 0  2  3 | 0  3  4 |
| **Sex**  Female  Male | 0  1 | 0  1 |
| **Hypertension**  No (systolic BP ≤140 mmHg and no  diagnosed hypertension/use of  antihypertensives  Yes (systolic BP >140 mmHg or  diagnosed hypertension/use of  antihypertensives | 0  2 | 0  2 |
| **BMI, kg/m^2^**  ≤30  >30 | 0  2 | 0  2 |
| **Hyperlipidemia**  No (no diagnosed hyperlipidemia/  use of lipid-lowering drugs)  Yes (diagnosed hyperlipidemia/  use of lipid-lowering drugs) | 0  2 | 0  1 |
| **Physical activity**  Yes  No | --  -- | --  -- |
| **APOE ε4 carrier**  No  Yes | --  -- | 0  2 |
| **Total** | 0–14 | 0–17 |

APOE=apolipoprotein E; BMI=body mass index; BP=blood pressure; CAIDE=Cardiovascular Risk Factors, Aging and Dementia

**Supplementary Table 2.** Baseline characteristics of the study population, by outcome (progression to AD dementia)

| Characteristics | Data available | All  (N=288) | Progression to AD dementia (N=91) | No progression to dementia (N=197) | P-value |
| --- | --- | --- | --- | --- | --- |
| **Demographics** |  |  |  |  |  |
| Age, years | 288 | 64.5 (9.1) | 67.9 (7.9) | 63.0 (9.1) | **<0.001** |
| Female | 288 | 165 (57.3%) | 55 (60.4%) | 110 (55.8%) | 0.46 |
| Education, years | 267 | 11.6 (3.8) | 11.6 (3.6) | 11.7 (3.9) | 0.84 |
| Follow-up time, years | 288 | 2.9 (1.9) | 3.0 (2.0) | 2.8 (1.9) | 0.57 |
| **Cognition** |  |  |  |  |  |
| MMSE score | 286 | 27.1 (2.6) | 27.0 (2.3) | 27.1 (2.7) | 0.68 |
| RAVLT, immediate recall score | 196 | 35.1 (9.9) | 30.7 (8.6) | 37.5 (9.7) | **<0.001** |
| RAVLT, delayed  recall score | 216 | 5.7 (3.3) | 4.2 (2.9) | 6.5 (3.3) | **<0.001** |
| **Biomarkers** |  |  |  |  |  |
| CSF Aβ42, pg/ml | 288 | 853.8 (298.5) | 670.5 (206.9) | 938.5 (296.7) | **<0.001** |
| CSF t-tau, pg/ml | 287 | 343.3 (190.0) | 470.0 (188.9) | 284.5 (159.7) | **<0.001** |
| CSF p-tau, pg/ml | 287 | 59.1 (25.8) | 71.4 (22.2) | 53.3 (25.4) | **<0.001** |
| Abnormal t-tau  (≥400 pg/ml) | 287 | 89 (31.0%) | 52 (57.1%) | 37 (18.9%) | **<0.001** |
| Abnormal p-tau  (≥80 pg/ml) | 287 | 59 (20.6%) | 30 (33.0%) | 29 (14.8%) | **<0.001** |
| CSF Aβ42/t-tau  ratio | 287 | 3.4 (2.0) | 1.7 (1.1) | 4.1 (2.0) | **<0.001** |
| CSF Aβ42/p-tau ratio | 287 | 18.0 (10.6) | 10.6 (5.5) | 21.4 (10.7) | **<0.001** |
| MTA score | 214 | 1.1 (0.8) | 1.3 (0.7) | 1.0 (0.8) | **0.004** |
| MTA score >1 | 214 | 72 (33.6%) | 33 (45.2%) | 39 (27.7%) | **0.01** |
| **Vascular factors** |  |  |  |  |  |
| Systolic BP, mmHg | 224 | 142.8 (19.0) | 147.5 (22.2) | 140.6 (17.1) | **0.01** |
| Diastolic BP, mmHg | 224 | 82.9 (10.1) | 83.4 (9.3) | 82.6 (10.5) | 0.57 |
| BMI, kg/m2 | 186 | 26.0 (4.1) | 25.5 (4.4) | 26.3 (4.0) | 0.25 |
| Current smoking | 263 | 39 (14.8%) | 13 (14.9%) | 26 (14.8%) | 0.97 |
| Hypertension | 288 | 114 (39.6%) | 38 (41.8%) | 76 (38.6%) | 0.61 |
| Hyperlipidemia | 288 | 81 (28.1%) | 28 (30.8%) | 53 (26.9%) | 0.50 |
| Diabetes | 288 | 48 (16.7%) | 15 (16.5%) | 33 (16.8%) | 0.96 |
| CAIDE risk score | 157 | 7.5 (2.5) | 7.8 (2.0) | 7.4 (2.6) | 0.35 |
| CAIDE risk score  with APOE | 86 | 9.3 (2.3) | 9.6 (2.0) | 9.2 (2.4) | 0.42 |
| **Other factors** |  |  |  |  |  |
| APOE ε4 carrier | 160 | 82 (51.3%) | 40 (71.4%) | 42 (40.4%) | **<0.001** |
| Family history of dementia | 277 | 109 (39.4%) | 37 (41.6%) | 72 (38.3%) | 0.60 |
| Cornell score | 226 | 6.1 (4.5) | 4.8 (3.4) | 6.6 (4.9) | **0.005** |

Data are mean (SD) or N (%). P-values are shown for comparisons between patients who developed AD dementia/did not develop any dementia. Hypertension and hyperlipidemia were defined as diagnosis of hypertension/hyperlipidemia and/or treatment with any antihypertensive/ lipid-lowering drug. Family history of dementia included at least one affected first-degree relative. AD=Alzheimer’s disease; APOE=apolipoprotein E; Aβ42=β-amyloid 1–42; BMI=body mass index; BP=blood pressure; CAIDE=Cardiovascular Risk Factors, Aging and Dementia; CSF=cerebrospinal fluid; MMSE=Mini-Mental State Examination; MTA=medial temporal lobe atrophy, visual rating; RAVLT=Rey Auditory Verbal Learning Test; p-tau=tau phosphorylated at threonine 181; t-tau=total tau.
